# Fractionating the Flavonoids in *Lonicerae japonicae* Flos and *Lonicerae flos* via Solvent Extraction Coupled with Automated Solid-Phase Extraction

**DOI:** 10.3390/foods13233861

**Published:** 2024-11-29

**Authors:** Lingyi Li, Shanbo Zhang, Bin Yu, Shao Quan Liu, Yancai Xiong

**Affiliations:** 1 School of Pharmacy, Hubei University of Science and Technology, Xianning 437100, China; 2 Department of Food Science and Technology, National University of Singapore, S14 Level 5, Science Drive 2, Singapore 117542, Singapore; e0729493@u.nus.edu (L.L.); zhangshanbo@u.nus.edu (S.Z.); 3 Mane SEA Pte Ltd., 3 Biopolis Drive, #07-17/18/19 Synapse, Singapore 138623, Singapore; gsgpbiy@gmail.com

**Keywords:** *Lonicerae japonicae* flos, *Lonicerae flos*, flavonoids, green chemistry, automated solid-phase extraction, antioxidant capacities

## Abstract

Due to the structural diversity of flavonoids in functional plant foods and the inherent limitations of existing techniques, it is important to develop a simple and green (environmentally friendly) method of extracting flavonoids from plant foods. In this study, a method involving solvent extraction followed by automated solid-phase extraction was developed for extracting flavonoids from *Lonicerae japonicae* flos (JYH) and *Lonicerae flos* (SYH), both of which are widely used functional plant-based foods in Asian countries. For the optimisation of the solvent extraction method, solvent concentration (0.0, 20.0, 40.0, 60.0, 80.0 and 100.0% (*v*/*v*) of ethanol–water solution), extraction temperature (40, 60 and 80 °C) and extraction time (15.0, 30.0, 60.0, 90.0 and 120.0 min) were evaluated via design of experiment after screening. For solid-phase extraction, five cartridges (Strata-X, InertSep RP-2, InertSep RP-C18, Bond Elut-ENV, Oasis Prime HLB) were evaluated and different elution steps were optimised to obtain high recoveries (79.69–140.67%) for eight target flavonoids, including rutin, isoquercetin and luteolin. Antioxidant capacity assays revealed that JYH samples demonstrated superior antioxidant potential compared to SYH. The optimised extraction method provides a valuable tool for industrial-scale flavonoid production.

## 1. Introduction

A number of plants are widely utilised in food and health industries for their purported health benefits [1]. Modern phytochemical investigations have revealed that these benefits are mainly attributed to bioactive ingredients, such as organic acids, iridoids, saponins and especially the flavonoids [2,3]. Flavonoids are secondary polyphenolic metabolites that are widely present in plant foods [4]. As a class of phenolic compounds, flavonoids are capable of donating protons to combine/react with free radicals generated by oxidation in the body to form stable products [5,6]. The strong antioxidant capacity of flavonoids can protect human organs from the harmful effects of free radicals while providing many other functional benefits, such as anti-inflammatory, antibacterial, anti-tumour, cardiovascular system protection and antiviral properties [7]. Hence, it is important to gain a better understanding of the flavonoids present in plant foods.

*Lonicerae japonicae* flos (flowers or flower buds of *Lonicera japonica* Thunberg, commonly known in Chinese as Jinyinhua, JYH), together with *Lonicerae flos* (flowers or flower buds of *Lonicera hypoglauca* Miquel, *Lonicera confusa* De Candolle or *Lonicera macrantha* (D.Don) Spreng, commonly known in Chinese as Shanyinhua, SYH), are two traditional edible plant-based foods widely cultivated in the Northern Hemisphere [8,9]. They have been consumed as a traditional functional tea beverage in Asia for thousands of years for its reported anti-inflammatory, antioxidant and antiviral benefits [10,11]. Recent phytochemical research has reported that these beneficial efficacies are mainly related to their bioactive components, especially the flavonoids [3], with variations in flavonoids compositions reported between them [12,13]. According to the differences in the structure of the heterocyclic C ring caused by varying extents of unsaturation and oxidation [14], flavonoids can be distinguished into different classes, such as flavonols, flavones, flavanones, anthocyanidins and isoflavones [15]. Each class has a wide variety of derivatives based on the quantity and nature of substituent groups attached to the flavonoid nucleus [16]. Due to the great assortment of flavonoids with varying chemical structures, polarities and concentrations, there is no standard procedure available to be applied for the extraction and separation of flavonoids [17]. Therefore, appropriate extraction methods would play an important role in the processing of flavonoids from plant materials [18].

Nevertheless, the extraction of flavonoids imposes various process limitations due to the complex plant matrices and the diverse types of metabolites in plant foods [2]. In addition, the most commonly used solvent extraction utilises mainly toxic organic solvents such as methanol [19] and hexane [20], which are not safe for human consumption. To ensure food-grade safety, additional steps are needed to remove toxic solvents, adding cost and complexity. Hence, it has become increasingly common to replace toxic solvents with food-grade ethanol and water due to their improved extraction efficiency, reduced toxicity and greater environmental sustainability [21,22]. Although the use of food-grade solvent extraction reduces the toxicity, it is necessary to tailor the extraction conditions to optimise the yield of target flavonoids. In previous studies, a few factors were found to be critical for the optimisation of solvent extraction, namely the ratio of solvents, temperature and extraction duration [23].

While optimised solvent extraction protocols allow for efficient recovery of flavonoids from the plant matrix, they do not provide sufficient selectivity for targeted flavonoid compounds [16]. To overcome this limitation, fractionation plays a crucial role in the extraction of bioactive ingredients from plant foods by improving the selectivity and functional potential of the resulting extracts [24,25]. Consequently, solid-phase extraction (SPE), a sample pre-treatment technique designed to purify and enrich extracts obtained from solvent extraction, was proposed as a critical fractionation step to enhance the separation and recovery of target compounds [26,27]. SPE involves using a solid adsorbent to first selectively adsorb target compounds in the liquid sample, separating them from the matrix and interfering compounds, then eluting them with a stronger solvent to achieve separation and purification of the target compounds [28,29,30]. Conventional offline SPE is typically carried out manually, involving multiple steps and significant time investment. This approach often leads to high artificial error rates, unstable recovery rates and variable method detection limits [31]. By contrast, automated SPE could reduce tedious sample manipulation to increase productivity and sample throughput [32,33].

Polymeric and (reversed) silica-based materials are the most commonly used sorbents for SPE [34]. Although reversed-phase silica sorbents offer good organic solvent resistance and mechanical stability, their low retention of polar compounds, instability at extreme pH and limited reusability make them less suitable for certain applications compared to polymeric sorbents [35,36]. In contrast, polymeric sorbents have the advantage of chemical stability and can extract compounds over a larger range of physicochemical characteristics [37,38]. Additionally, flavonoids in plant foods, due to the complexity and diversity of their chemical structures, have different hydrophilic capacities and polarities, which necessitate the need to select a suitable sorbent that allows simultaneous extraction of a wide range of compounds of different natures. Thus, in this study, five polymeric reversed-phase cartridges were assessed to obtain an optimal sorbent for the extraction of flavonoids in plant foods (JYH and SYH). Moreover, similar to sorbents, the concentration of the solvent has different effects on the extraction of flavonoids with different polarities [37], so a concentration gradient of elution solvents was designed in this study.

In this study, flavonoids were extracted from JYH and SYH. Solvent extraction optimisation was first carried out with screening experiments followed by a detailed design of experiment (DOE) workflow. An automated SPE gradient elution programme was then used to further fractionate and purify the flavonoids extract. Finally, total flavonoid contents (TFC) and antioxidant capacity assays were performed to investigate the contribution of each SPE fraction to their antioxidant properties.

## 2. Materials and Methods

### 2.1. Plant Materials and Chemicals

JYH was purchased from Shandong Province, China, and SYH was purchased from Hunan Province, China. The JYH and SYH were stored in a dry, cool and dark environment. The stems of JYH and SYH were used for this study. Eight major flavonoids were selected based on a study [9], namely rutin, hyperoside, isoquercetin, cynaroside, lonicerin, nicotiflorin, rhoifolin and luteolin (Table 1). Analytical-grade standards (>98% purity) for each flavonoid were purchased from Chemfaces, Wuhan, China. Acetonitrile (ACN) (Fisher Scientific Co., Waltham, MA, USA), water (Fisher Scientific Co., Waltham, MA, USA) and formic acid (Merck, Darmstadt, Germany) were high performance liquid chromatography (HPLC)-grade solvents utilised for the HPLC studies. Food-grade ethanol was obtained from Mane Co., Ltd., Bangkok, Thailand. The chemicals used for TFC analysis were sodium nitrite, aluminium chloride and sodium hydroxide purchased from Sigma Aldrich(St. Louis, MI, USA. Additionally, 2,2-azinobis (3-ethylbenzothiazoline-6-sulfonic acid) diammonium salt (ABTS), 2,2’-azobis (2-methylpropionamidine) dihydrochloride (AAPH), potassium persulfate, fluorescein disodium, dipotassium hydrogen phosphate and 6-hydroxy-2,5,7,8-tetramethylchroman-2-carboxylic acid (Trolox) were purchased from Sigma Aldrich (St. Louis, MI, USA) for the antioxidant capacity assay.

### 2.2. HPLC Analysis

The eight flavonoids were analysed on a 1260 infinity II HPLC-UV (Agilent, Santa Clara, CA, USA). The separation was conducted on a Poroshell 120 EC-C18 column (150 mm × 3.0 mm × 2.7 µm, Agilent, Santa Clara, CA, USA) at 40 °C. The mobile phase was composed of water containing 0.1% (*v*/*v*) formic acid (A) and ACN (B). The gradient elution programme was set based on Su et al. [39] due to the suitability of this range for polyphenol compounds: 0–2 min, 5% B; 2–8 min, 5–13% B; 8–22 min, 13–28% B; 22–25 min, 28–95% B; 25–28 min, 95% B; 28–28.1 min, 95–5% B; 28.1–35 min, 5% B. The flow rate was 0.40 mL/min, and the injected volume was 20 μL. The UV spectrum was recorded between 190 and 400 nm, and absorbance at λ = 350 nm was recorded. All selected flavonoids exhibited maximum absorbance at 350 nm.

### 2.3. HPLC Method Validation

The accuracy of the HPLC method was first validated to assure the reliability of the generated results. The linearity was determined with six different concentrations of individual reference compounds. Calibration curves were established with cynaroside (0.050–100.000 μg/mL), rutin, isoquercetin, lonicerin, nicotiflorin (0.100–50.000 μg/mL), hyperoside, rhoifolin and luteolin (0.100–100.000 μg/mL). The correlation coefficient (R^2^) was calculated for each flavonoid using a regression equation.

The sensitivity of the method was determined by the limit of detection (LOD) and the limit of quantification (LOQ). The Eurachem Guide was used to determine the LODs and LOQs [40]. Repeatability (intra-day precision) and reproducibility (inter-day precision) were determined in triplicate by injecting a standard solution of the minimum concentration of the eight flavonoids for analysis on three consecutive days [41]. Intra-day relative standard deviation (RSD) was calculated as the mean of RSD for the three injections within a single day, while inter-day RSD was calculated as the RSD of injections across three consecutive days (three injections each day).

### 2.4. Optimisation of Solvent Extraction

#### 2.4.1. Extraction Screening

Ethanol concentration, extraction time and extraction temperature were selected as the parameters for extraction screening. The concentration of the ethanol–water solution and extraction temperature were optimised under a fixed extraction time treatment, and the extraction time was optimised under a fixed ethanol concentration and extraction temperature. One gram (1.000 g) of JYH was weighed (Mettler Toledo, Greifensee, Switzerland) and added into 10 mL of an ethanol–water mixture containing 0.0, 20.0, 40.0, 60.0, 80.0 or 100.0% (*v*/*v*) ethanol. Each sample was extracted for 60 min at a designated temperature (40, 60 and 80 °C). Another 1.000 g of sample was added into 10 mL of an ethanol–water mixture and extracted at 40.0% (*v*/*v*) ethanol and 40 °C for varying extraction times (15, 30, 60, 90 and 120 min). After extraction, all the mixtures were centrifuged at 8000 rpm and 4 °C for 10 min (Eppendorf centrifuge 5804 R, Hamburg, Germany). An aliquot of 2 mL of the supernatant was then filtered through a 0.22 µm regenerated cellulose membrane (Agilent, Santa Clara, CA, USA) prior to HPLC analysis. After analysis, three key points (high, medium, low) of parameters were selected for optimisation.

#### 2.4.2. Extraction Parameters Optimisation

Extraction parameters (time and temperature) for the solvent extraction method were optimised using response surface methodology (RSM) via a central composite design (CCD). For an experimental design with two factors, the quadratic model can be expressed by the following equation:

Y = a_0_ + a_1_A_1_ + a_2_B_1_ + a_3_A^2^ + a_4_B^2^ + a_5_A_1_B_1_ (1)

where Y is the predicted percentage value of concentration/response; A is the extraction ethanol concentration (*v*/*v* %); B is the extraction time (min); and a_0_–a_5_ are the coefficient values obtained through multiple linear regression using Minitab 19 software.

The CCD approach utilised three groups of design points (each factor was set at five levels) as described in Table 2a,b to model the relationship between extraction parameters (extraction ethanol concentration (*v*/*v* %) and extraction time (min)) under 40 °C and recovery with a minimal number of 13 experimental runs. The parameter levels were 20.0% (low) to 60.0% (high) and 30.0 min (low) to 90.0 min (high). After optimisation, the same parameters were used to extract flavonoids from SYH for further analysis.

### 2.5. SPE Method Development

An automated SPE apparatus (Freestyle SPE module, LC Tech, Obertaufkirchen, Germany) was used to extract flavonoids in the JYH and SYH extracts obtained above.

JYH extracts from Section 2.4 were used for SPE method development. Five types of cartridges were prepared for comparison, namely Strata-X (Phenomenex, Torrance, CA, USA), InertSep RP-2 (GL Sciences, Tokyo, Japan), InertSep RP-C18 (GL Sciences, Tokyo, Japan), Bond Elut-ENV (Agilent Technologies, Santa Clara, CA, USA) and Oasis Prime HLB (Waters Corporation, Massachusetts, USA). For consistency, the size of all cartridges used was the same (500 mg/6 mL).

**Figure 1:**
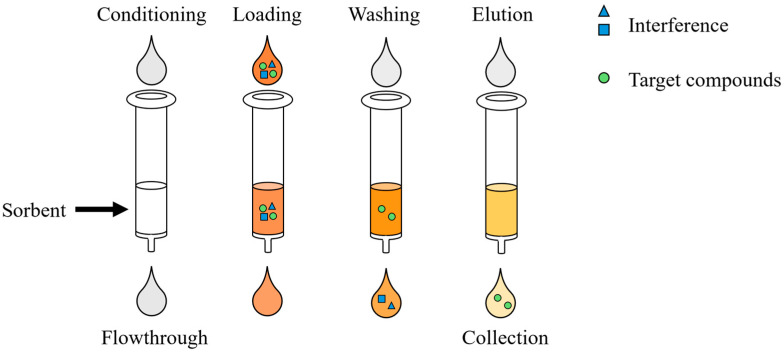
The elution programme for cartridge comparison.

**Figure 2:**
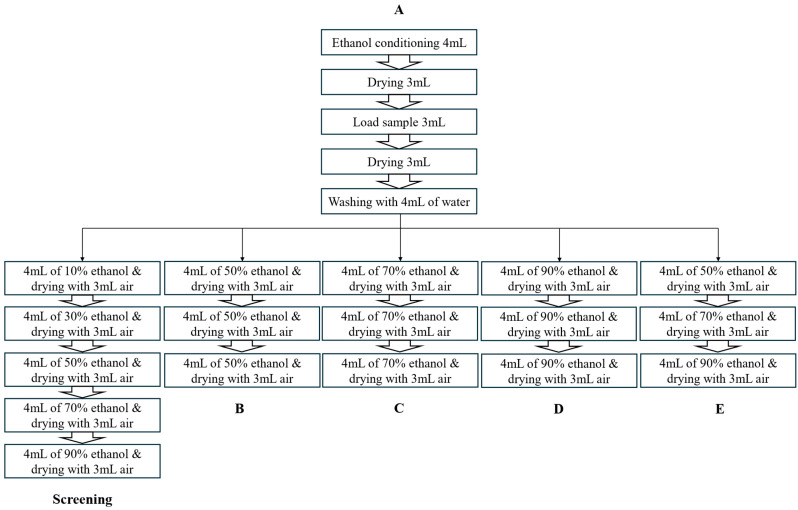
The specific parameters of each elution programme.

Figure 1 illustrates the elution programme for cartridge comparison, and the specific parameters of each step are presented in Figure 2. Cartridges were initially conditioned with 4 mL of ethanol and sequentially with 4 mL of water at a flow rate of 5 mL/min. A volume of 40 mL of the extracted sample was then loaded onto the cartridge. Each fraction was obtained by adding 4 mL of 10, 30, 50, 70 and 90% ethanol sequentially to elute the sample. Drying was performed in between each step. The extracted sample and the subsequent eluted fractions were analysed using HPLC. For the optimisation of the elution programme, elution steps were conducted by following the flowchart shown in Figure 2.

### 2.6. TFC Analysis and Antioxidant Capacity Assays of JYH and SYH Extracts

The 96-well polystyrene microplates and the corresponding covers obtained from VWR International Inc. (Bridgeport, NJ, USA) were used to measure TFC and antioxidant capacity. A Biotek Synergy HTX Multi-Mode Reader with Auto Pipette (Agilent, Santa Clara, CA, USA) was used for absorbance measurements.

#### 2.6.1. TFC Analysis

TFC was measured according to the aluminium complex colorimetric method with some modifications [42]. A volume of 60 μL of each SPE fraction was mixed with 10 μL of 5% (*v*/*v*) sodium nitrite solution and incubated at room temperature for 6 min. Then, 10 μL of 10% (*v*/*v*) aluminium nitrate solution was added and the solution was vortexed thoroughly. After another 6 min of incubation, 100 μL of 4% (*w*/*w*) sodium hydroxide solution was added followed by an additional 15 min of incubation. The absorbance of the resultant solution was measured at 510 nm. Stock solutions of the extracts were prepared at a concentration of 4 mg/mL in methanol, followed by serial dilution to yield solutions of varying concentrations (0.25 mg/mL, 0.50 mg/mL, 0.75 mg/mL and 1.00 mg/mL). Subsequently, 1 mL of each quercetin concentration was added to a test tube containing 4 mL of distilled water. A standard calibration curve (y = 0.0086x + 0.0378; R^2^ = 0.9983) was constructed using quercetin hydrate as a reference and the flavonoids concentration was reported in terms of quercetin equivalents (QE) (μg QE/mL).

#### 2.6.2. Antioxidant Capacity Assays

The antioxidant capacity assays were performed by measuring the scavenging activity of ABTS radical cations and the oxygen radical absorbance capacity (ORAC). ABTS solution was prepared by mixing equal parts of 7.00 mM ABTS solution and 2.45 mM potassium persulfate solution followed by incubation in darkness at room temperature for 16 h. A volume of 10 μL of the experimental blank, Trolox solution (25–500 μg/mL) and diluted JYH and SYH fractions were pipetted into individual wells in a 96-well plate. An aliquot (190 μL) of the ABTS solution was then added into each well. The well plate was incubated in darkness for 10 min. The absorbance at 734 nm was then recorded for each sample. An external standard curve was plotted using Trolox with R^2^ > 0.99. The ORAC assay was carried out on a fluorescence microplate reader with an excitation wavelength of 485 nm and an emission wavelength of 525 nm. The temperature of the incubator was set to 37 °C. The procedure was based on the modified ORAC method [43]. AAPH was used as the peroxyl generator. Fluorescein solution (150 μL) (4.19 × 10^−3^ mM), 25 μL of AAPH and 25 μL of sample were mixed in each well. The fluorescence readings were taken every 2 min for 2 h, and the area under the curve (AUC) was calculated for each sample. Trolox was also used to establish the calibration curve with a linear range of 0.78 to 62.50 μM. All dilutions were carried out using 75.00 mM K_2_HPO_4_·3H_2_O buffer at pH 7.38. Data are expressed as Trolox equivalents (TE) per mL of JYH and SYH extracts (μg TE/mL).

### 2.7. Data Processing and Statistical Analysis

All experimental data were acquired in triplicate, and the mean values and standard deviations were reported. RSM was conducted using Minitab 19 software. Data visualising was carried out using Origin 2022 SR1 (OriginLab, Northampton, MA, USA).

## 3. Results and Discussion

### 3.1. Validation of HPLC Method

The HPLC method was first adapted from previous literature [39] and validated for detection of the eight targeted flavonoids to ensure good accuracy and precision. The linear range, correlation coefficient, LODs, LOQs, repeatability and reproducibility were all determined through a series of studies (Table 3). The standard curve linearities for eight flavonoids were all >0.9993, indicating good linearity across a wide concentration range. The Eurachem Guide was used to determine the LODs and LOQs. The LODs of the eight flavonoids varied from 0.008 to 0.022 μg/mL, while the LOQs ranged from 0.025 to 0.075 μg/mL. The method showed good repeatability and reproducibility. The RSDs for peak area and retention time of the eight flavonoids for intra-day repeatability were 0.49–1.34% and 0.14–0.22%, respectively, while the inter-day RSDs for peak area and retention time were 0.32–2.39% and 0.01–0.07%, respectively. The optimised method was then applied for the identification and quantification of the flavonoids in the JYH and SYH samples.

### 3.2. Screening of Single Factors on Flavonoids Yields

**Figure 3:**
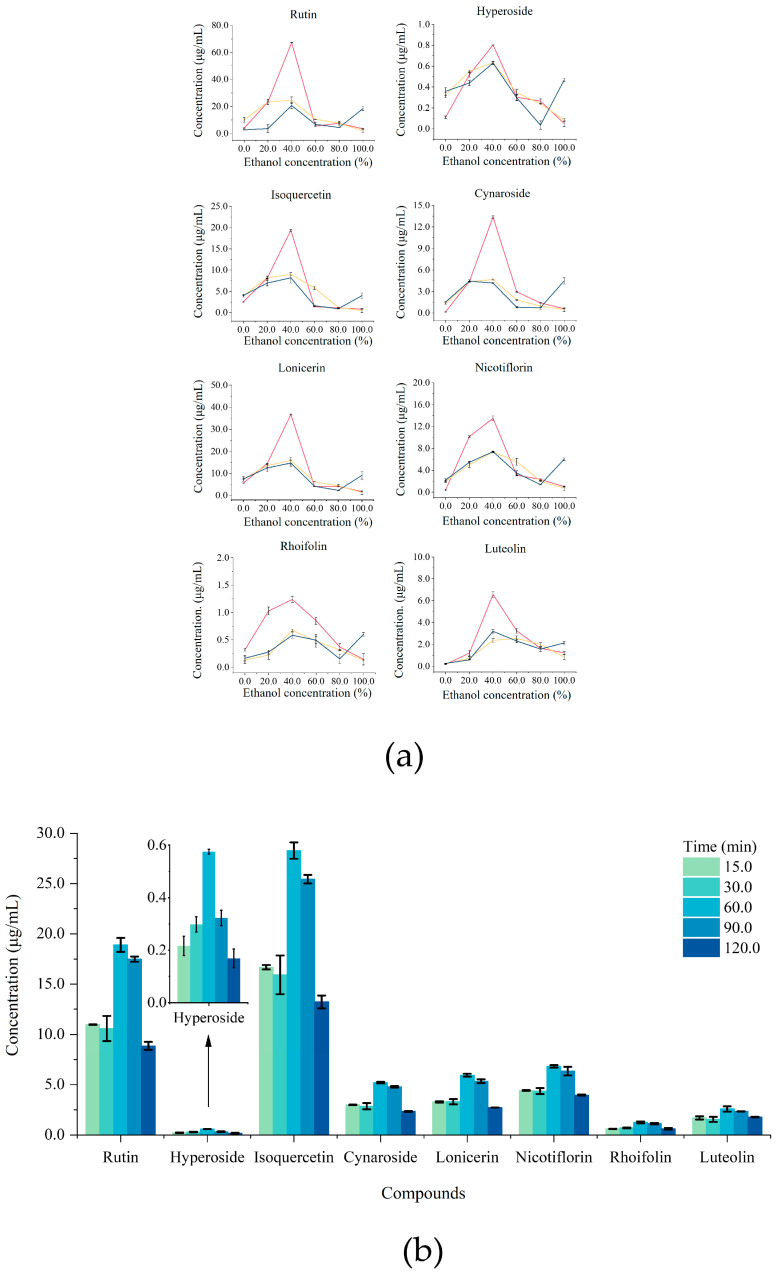
Effects of ethanol concentration, extraction temperature and extraction time on the extraction yields of flavonoids from JYH: (**a**) Effects of ethanol concentration and extraction temperature on the extraction yields of flavonoids from JYH. (40 °C (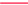), 60 °C (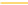), 80 °C (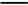)); (**b**) effects of extraction time on the extraction yield of flavonoids from JYH (For interpretation of the references to colour in this figure legend, the reader is referred to the Web version of this article).

After reviewing the literature, a range of ethanol concentrations and extraction temperatures was selected as our investigated conditions. The influence of ethanol concentration and extraction temperature on the yield of eight flavonoids is shown in Figure 3a. With a constant extraction time of 60 min, the yield of flavonoids was observed to increase with increasing ethanol concentration until it reached the highest abundance at 40.0% ethanol concentration and then decreased. Therefore, 20.0–60.0% was selected as the DOE optimisation range for ethanol concentration, and 20.0, 40.0 and 60.0% ethanol were chosen as low, medium and high points for optimisation using DOE. For temperatures, there was no clear relationship between temperature and yield. However, in general, extraction efficiency was the best at 40 °C (Figure 3a). Besides, it was worth noting that high temperature (80 °C) combined with high ethanol concentration (above 80.0%) could cause other impurities to be dissolved into the supernatant, leading to an apparent increase in the concentration of all compounds. This phenomenon was also demonstrated by Zhong et al. [44]. According to Figure 3b, similar to ethanol concentration, excessive extraction time (causing flavonoids decomposition) or insufficient extraction time (poor extraction) could result in a low concentration of flavonoids [45]. The highest yield of flavonoids was achieved at 60.0 min. Thus, extraction time of 30.0–90.0 min was chosen as DOE optimisation range, and 30.0, 60.0 and 90.0 min were set as our DOE optimisation parameters.

### 3.3. Optimisation of Solvent Extraction After CCD

**Figure 4:**
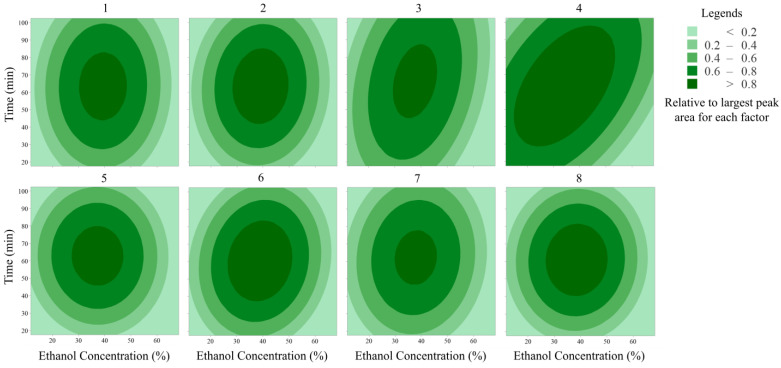
2D contour plots of the yields of eight flavonoids.

The 2D contour plots of the yield of eight flavonoids and estimated response surface for the extraction time and ethanol concentration are shown in Figure 4. The fitting results are presented in Table 4. In the 2D contour plots, if the contour lines are spaced close to each other, then the values change rapidly; if the contour lines are spaced far apart, then the values change slowly. From Figure 4, it can be seen that the plots of rutin, hyperoside, lonicerin, nicotiflorin, rhoifolin and luteolin are similar. Extraction time and ethanol concentration had equal influences on the solvent extraction of these compounds. For isoquercetin, ethanol concentration had a greater effect than extraction time on the yield. After RSM fitting, the optimised conditions of the eight flavonoids that are obtained using Minitab 19 software ranged from 34.0 to 39.3% of ethanol and 60.4 to 66.4 min of extraction time. The mean for each parameter was calculated, resulting in a final optimal ethanol concentration of 37.8% (*v*/*v*) and an extraction time of 63.0 min. Similar results were reported by Zhuang et al. [46] for flavonoids extracted from *Nicandra physalodes*. Furthermore, Youn et al. [47] pointed out that in the range of 30.0 to 40.0% (*v*/*v*) ethanol, the antioxidant capacity of total polyphenols and flavonoids was the highest. Therefore, to maintain the bioactivity of flavonoids extracts in JYH, the optimal conditions (37.8% (*v*/*v*) and 63.0 min) were selected to obtain JYH extracts for SPE.

### 3.4. Selection of SPE Cartridge and Elution Solvent Concentration

After determination of optimal conditions of solvent extraction, SPE was performed for fractionation and enrichment of these eight flavonoids. The first parameter that should be considered in SPE is cartridge selection, specifically the cartridge performance for every compounds. The recovery of each flavonoid was calculated through dividing by the amount of each flavonoid in the extract before SPE. The recovery results are shown in Table 5. RP-C18, RP-2 and ENV cartridges showed poor performance on the recovery of all eight flavonoids. In comparison, Strata-X and HLB cartridges exhibited good recoveries for relatively hydrophilic flavonoids. The log *p* value, which represents the octanol–water partition coefficient, is a measure of a compound’s hydrophobicity [48]. Compounds with negative log *p* values tend to be hydrophilic, whereas those with positive log *p* values are hydrophobic. In this study, the log *p* values of rutin, lonicerin, nicotiflorin and rhoifolin were all below zero, indicating their relatively hydrophilic nature (Table 1). This characteristic is consistent with their high recoveries (80–130%) when extracted with hydrophilic–lipophilic balanced (HLB) cartridges in solid-phase extraction (SPE). In comparison, Strata-X cartridges yielded superior recovery only for luteolin, suggesting that HLB cartridges are more suitable for extracting relatively hydrophilic flavonoids. A similar result was reported by Sergiel et al. [49]. Strata-X recovered more than 90% of luteolin (log *p* = 1.5) in JYH. The higher recovery of Strata-X could be explained by the sorbent with functionalised polymers, which were more likely to form effective adsorption with hydrophilic flavonoids (rutin, lonicerin, nicotiflorin and rhoifolin) through π-π bonding and hydrogen bonding [17]. Based on the observed performance of the five cartridges, the Strata-X cartridge was selected for further optimisation steps.

(2) $$\mathrm{Recovery}\;(\%)=\frac{\left(concentration\;of\;each\;flavonoid\;in\;fractionations\;of\;10,30,50,70,\;and\;90\%\;ethanol\right)}{concentration\;of\;each\;flavonoid\;in\;the\;mixture\;before\;SPE}\times 100\%$$

The concentrations of flavonoids in each SPE fraction are listed in Table 6. The fraction with the highest yields was eluted with 50% ethanol, except for isoquercetin and luteolin. The main components of the 50% ethanol fraction were rutin (52.58 ± 1.43 µg/g), isoquercetin (31.89 ± 1.48 µg/g), cynaroside (12.07 ± 0.44 µg/g), lonicerin (38.34 ± 1.22 µg/g) and nicotiflorin (38.31 ± 0.44 µg/g). Notably, hyperoside, rhoifolin and luteolin were not detected in eluted fractions with ethanol concentrations lower than 50%. Instead, hyperoside and rhoifolin demonstrated higher yields in the eluted fraction with 70% ethanol (1.00 ± 0.04 µg/g and 2.12 ± 0.30 µg/g, respectively), and luteolin was only detected in the 90% ethanol fraction. Therefore, 50, 70 and 90% ethanol were selected for further optimisation of the elution programme. 

### 3.5. Optimisation of Elution Programme

**Figure 5:**
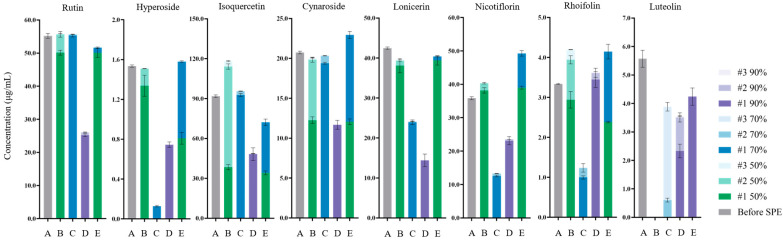
Optimisation of the SPE elution programme for simultaneous fractionation of flavonoids. Amount: concentration of each flavonoid in each fraction. The letters A, B, C, D and E correspond to different elution protocols indicated in Figure 2. Before SPE (A): the amount of eight selected flavonoids before SPE; 50% × 3 (B): the procedure using four millilitres of 50% ethanol to elute the sample three times; 70% × 3 (C): the procedure using four millilitres of 70% ethanol to elute the sample three times; 90% × 3 (D): the procedure using four millilitres of 90% ethanol to elute the sample three times; 50% × 1 and 70% × 1 and 90% × 1 (E): this represents the sample that was eluted by four millilitres of 50% ethanol followed by four milliliters of 70% ethanol followed by four millilitres of 90% ethanol.

The elution programme was subsequently redesigned and simplified, using different proportions of 50, 70 and 90% ethanol for the elution step (Figure 5). Rutin, hyperoside, lonicerin, nicotiflorin and rhoifolin had better elution recoveries when the first elution step was performed using 50% ethanol as compared to 70% and 90% ethanol. Hence, 50% ethanol was chosen as the first elution step. The remaining compounds (cynaroside, luteolin and isoquercetin) required a higher elution strength (70 or 90% ethanol). A second elution step using 70% ethanol successfully eluted cyanroside and isoquercetin, while luteolin was subsequently eluted using 90% ethanol. Compared to multiple extractions with the same concentration of ethanol, a series of elution steps with increasing elution strength (50% × 1, 70% × 1, 90% × 1) resulted in a higher yield of flavonoids. These results were also consistent with the log *p* values of these flavonoids. Rutin, lonicerin, nicotiflorin and rhoifolin are relatively hydrophilic, while cynaroside, isoquercetin and luteolin are relatively hydrophobic and thus, the latter ones required a higher concentration of ethanol for the elution. As such, a combination of multiple eluents would aid in recovering these flavonoids of varying polarities.

Based on the above results, a new elution programme was designed with three elution steps: first using 4 mL of 50% ethanol, followed by 4 mL of 70% ethanol and finally 4 mL of 90% ethanol (elution protocol E). The recoveries of all flavonoids were above 80%. Due to the simplicity and feasibility of the elution programme, it could be used to simultaneously fractionate targeted flavonoids with various properties from authentic JYH samples.

### 3.6. TFC and Antioxidant Capacity of JYH and SYH Samples

The optimised solvent extraction and SPE conditions were applied to JYH and SYH and the TFC and antioxidant capacities (ORAC and ABTS) of the different fractions were measured. Table 7 shows the quantities of eight selected flavonoids and antioxidant capacities in different elution fractions for both JYH and SYH. From the results, the flavonoid constituents were different in different elution fractions and species. In general, JYH was abundant in rutin (54.78 µg/g), lonicerin (40.15 µg/g) and nicotiflorin (49.15 µg/g), while SYH was rich in isoquercetin (90.56 ± 0.75 µg/g) and rhoifolin (9.82 ± 0.09 µg/g). Among different fractions after gradient elution SPE, it was found that JYH 50%, JYH 70%, SYH 50% and SYH 70% were relatively high in isoquercetin (Table 7), and these fractions recorded greater antioxidant capacities. Comparatively, JYH 90%, and SYH 90% had lower abundances of the aforementioned flavonoids and consequently lower antioxidant capacities. For the four fractions with high antioxidant capacities, isoquercetin may be the key flavonoid distinguishing these fractions. From SYH 70% to SYH 90%, the significant decrease in antioxidant capacity may be attributed to the decrease in isoquercetin. In conclusion, a combination of extraction and fractionation methods can yield a high-purity, flavonoid-rich fraction. Through this approach, it would be convenient and efficient to select high-quality JYH and SYH and isolate desirable fractions.

## 4. Conclusions

In this study, CCD was successfully used to optimise the extraction conditions of eight flavonoids from JYH and SYH samples. The optimised conditions were 37.8% (*v*/*v*) ethanol for the extraction solvent and 63.0 min of extraction time. Subsequently, automated SPE was optimised to fractionate the targeted flavonoids with substantial recoveries (79.69–140.67%) from the complex matrices. The final elution protocol involved three 4 mL elutions through a Strata-X cartridge using 50%, 70% and 90% ethanol in sequential order. All eight flavonoids from JYH and SYH were enriched and fractionated into different fractions exhibiting similar trends. For instance, rutin, lonicerin and nicotiflorin from both JYH and SYH were predominantly enriched in the 50% fraction, whereas luteolin from both JYH and SYH was only enriched in the 90% fraction. In conclusion, a high throughput automated SPE coupled with green and simple solvent extraction procedures was developed. This study’s findings have significant implications for enhanced extraction efficiency, quality control and standardisation in plant foods. This methodology could be further expanded and scaled to industrial standards for food processing and high recovery extraction of flavonoid compounds from plant materials.

## Figures and Tables

**Table 1:** Basic information on eight selected flavonoids.

No.	Compounds	Chemical Class	Chemical Structure	Log *p*	Hydrogen Bond Donor Count	CAS Number
1	Rutin	Flavonol	C_27_H_30_O_16_	−0.7	10	153-18-4
2	Hyperoside	Flavonol	C_21_H_20_O_12_	0.1	8	482-36-0
3	Isoquercetin	Flavonol	C_21_H_20_O_12_	0.1	8	482-35-9
4	Cynaroside	Flavone	C_21_H_20_O_11_	0.2	7	5373-11-5
5	Lonicerin	Flavone	C_27_H_30_O_15_	−0.7	9	25694-72-8
6	Nicotiflorin	Flavonol	C_27_H_30_O_15_	−1.1	9	17650-84-9
7	Rhoifolin	Flavone	C_27_H_30_O_14_	−1.0	8	17306-46-6
8	Luteolin	Flavone	C_15_H_10_O_6_	1.5	4	491-70-3

**Table 2:** (**a**) Experimental parameters and their levels for the optimisation of extraction time and ethanol concentration. (**b**) Central composite design matrix for extraction ethanol concentration and time.

(**a**)						
**Variables**		**Level**			**Star Points (*α* = 1.41)**	
		**Low (−1)**	**Central (0)**	**High (+1)**	**−*α***	**+*α***
Extraction ethanol concentration (*v*/*v* %)		20.0	40.0	60.0	11.7	68.3
Extraction time (min)		30.0	60.0	90.0	17.6	102.4
(**b**)						
**No.**	**Extraction Ethanol Concentration (*v*/*v* %)**				**Extraction Time (min)**	
1	20.0				30.0	
2	20.0				90.0	
3	60.0				30.0	
4	60.0				90.0	
5	40.0				17.6	
6	40.0				102.4	
7	11.7				60.0	
8	68.3				60.0	
9	40.0				60.0	
10	40.0				60.0	
11	40.0				60.0	
12	40.0				60.0	
13	40.0				60.0	

**Table 3:** Linear range, correlation coefficient, LODs, LOQs, repeatability and reproducibility of HPLC method.

No.	Compounds	Calibration Equation	Coefficient of Determination (R^2^)	Linear Range (µg/mL)	LOD (µg/mL)	LOQ (µg/mL)	% RSD Intra-Day (n = 3) ^a^		% RSD Inter-Day (n = 3) ^b^	
							Peak Area (%)	Retention Time (%)	Peak Area (%)	Retention Time (%)
1	Rutin	y = 61.261x + 3.073	0.9997	0.100–50.000	0.012	0.042	0.85	0.21	0.33	0.01
2	Hyperoside	y = 56.700x + 2.230	0.9999	0.100–100.000	0.008	0.026	0.53	0.15	0.41	0.05
3	Isoquercetin	y = 21.834x + 4.098	0.9997	0.100–50.000	0.015	0.049	1.02	0.22	0.68	0.01
4	Cynaroside	y = 95.829x + 8.330	0.9999	0.050–100.000	0.008	0.027	0.59	0.14	2.38	0.07
5	Lonicerin	y = 36.682x + 3.050	0.9994	0.100–50.000	0.009	0.031	0.65	0.22	0.47	0.03
6	Nicotiflorin	y = 46.461x + 1.037	0.9993	0.100–50.000	0.008	0.025	0.49	0.20	0.32	0.03
7	Rhoifolin	y = 55.123x + 6.728	0.9999	0.100–100.000	0.022	0.075	1.34	0.22	0.42	0.01
8	Luteolin	y = 45.115x + 0.233	0.9998	0.100–100.000	0.008	0.027	0.55	0.19	2.39	0.01

^a^ Repeatability (intra-day), n = 3, calculation based on the mean of RSD (three injections within same day). ^b^ Reproducibility (inter-day), n = 3, calculation based on the mean of RSD (three injections each day across the three days).

**Table 4:** RSM estimated optimal conditions for each flavonoid.

No.	Compounds	Optimised Extraction Ethanol Concentration (*v*/*v* %)	Optimised Extraction Time (min)
1	Rutin	39.3	63.9
2	Hyperoside	39.0	63.9
3	Isoquercetin	37.4	66.4
4	Cynaroside	34.0	63.0
5	Lonicerin	37.3	63.0
6	Nicotiflorin	38.9	60.4
7	Rhoifolin	38.0	62.1
8	Luteolin	38.5	61.3
	Mean	37.8	63.0

**Table 5:** Comparison of SPE cartridges of RP-C18, Strata, HLB, RP-2 and ENV based on recovery of eight selected flavonoids under the cartridge screening gradient elution programme.

No.	Compounds	Recovery (%) ^a^				
		RP-C18	Strata-X	HLB	RP-2	ENV
1	Rutin	13.89 ± 0.51	105.39 ± 1.58	98.06 ± 5.51	28.43 ± 1.49	51.48 ± 0.57
2	Hyperoside	44.69 ± 8.09	140.67 ± 2.62	149.20 ± 5.26	80.15 ± 6.15	31.92 ± 1.56
3	Isoquercetin	18.51 ± 0.53	94.53 ± 1.44	97.87 ± 9.74	49.42 ± 5.34	77.07 ± 0.93
4	Cynaroside	17.13 ± 0.35	107.01 ± 4.19	100.74 ± 9.82	36.36 ± 1.42	76.29 ± 1.16
5	Lonicerin	14.78 ± 0.49	85.78 ± 1.60	78.19 ± 9.10	19.42 ± 1.02	46.21 ± 0.76
6	Nicotiflorin	9.05 ± 0.19	131.10 ± 4.67	116.53 ± 5.52	15.59 ± 0.97	1.27 ± 0.11
7	Rhoifolin	30.75 ± 0.47	86.63 ± 6.47	98.00 ± 4.91	51.96 ± 4.31	185.70 ± 6.63
8	Luteolin	64.19 ± 3.36	79.69 ± 3.03	25.38 ± 8.07	27.81 ± 2.04	95.30 ± 1.49

Values in each cell are presented as mean ± standard deviation. ^a^ Recovery was calculated based on HPLC data under the following formula.

**Table 6:** Collected flavonoids and recovery in each fraction using Strata-X cartridge after automated SPE.

No.	Compounds	Flowthrough (µg/g)	Washing (µg/g)	10% Ethanol (µg/g)	30% Ethanol (µg/g)	50% Ethanol (µg/g)	70% Ethanol (µg/g)	90% Ethanol (µg/g)	Recovery (%) ^a^
1	Rutin	-	-	-	4.78 ± 0.63	52.58 ± 1.43	1.84 ± 0.23	0.36 ± 0.01	105.39 ± 1.58
2	Hyperoside	-	-	-	-	1.04 ± 0.10	1.00 ± 0.04	0.25 ± 0.01	140.67 ± 2.62
3	Isoquercetin	-	-	-	2.86 ± 0.34	31.89 ± 1.48	35.62 ± 2.38	0.47 ± 0.02	94.53 ± 1.44
4	Cynaroside	-	-	-	1.02 ± 0.03	12.07 ± 0.44	10.90 ± 0.51	-	107.01 ± 4.19
5	Lonicerin	-	-	-	1.92 ± 0.06	38.34 ± 1.22	1.40 ± 0.17	0.41 ± 0.01	85.78 ± 1.60
6	Nicotiflorin	-	-	-	1.25 ± 0.57	38.31 ± 0.44	10.40 ± 0.83	0.44 ± 0.01	131.10 ± 4.67
7	Rhoifolin	-	-	-	-	2.73 ± 0.14	2.12 ± 0.30	0.35 ± 0.01	86.63 ± 6.47
8	Luteolin	-	-	-	-	-	-	4.68 ± 0.31	79.69 ± 3.03

Values in each cell are presented as mean ± standard deviation. Microgram of each flavonoid in per gram of JYH samples. ‘-’ means compounds were not detected. After calculating the *p*-value between each two values using *t*-test, they were significantly different (*p* < 0.05) from each other. ^a^ Recovery was calculated based on HPLC data under the following formula.

**Table 7:** The quantities of eight selected flavonoids and antioxidant capacities in JYH and SYH samples in different fractions using the optimised solvent extraction and SPE conditions.

No.	Compounds	JYH ^a^			SYH ^b^		
		50% Fraction (µg/g)	70% Fraction (µg/g)	90% Fraction (µg/g)	50% Fraction (µg/g)	70% Fraction (µg/g)	90% Fraction (µg/g)
1	Rutin	52.58 ± 1.43	1.84 ± 0.23	0.36 ± 0.01	0.81 ± 0.05	-	-
2	Hyperoside	1.04 ± 0.10	1.00 ± 0.04	0.25 ± 0.01	0.55 ± 0.02	-	-
3	Isoquercetin	31.89 ± 1.48	35.62 ± 2.38	0.47 ± 0.02	50.43 ± 0.90	40.73 ± 1.97	-
4	Cynaroside	12.07 ± 0.44	10.90 ± 0.51	-	2.76 ± 0.17	0.43 ± 0.01	-
5	Lonicerin	38.34 ± 1.22	1.40 ± 0.17	0.41 ± 0.01	2.64 ± 0.05	-	-
6	Nicotiflorin	38.31 ± 0.44	10.40 ± 0.83	0.44 ± 0.01	0.50 ± 0.01	-	-
7	Rhoifolin	2.73 ± 0.14	2.12 ± 0.30	0.35 ± 0.01	7.90 ± 0.01	2.64 ± 0.09	-
8	Luteolin	-	-	4.68 ± 0.31	-	-	0.50 ± 0.01
	Antioxidant Capacity						
9	TFC ^c^	1740.00 ± 62.92	1005.28 ± 44.55	84.39 ± 4.28	1905.28 ± 85.53	992.78 ± 46.09	78.83 ± 2.50
10	ORAC ^d^	4656.81 ± 107.91	4295.68 ± 96.91	2282.76 ± 214.00	4881.59 ± 21.57	4199.65 ± 12.31	2496.02 ± 125.87
11	ABTS ^d^	798.72 ± 26.55	510.67 ± 9.96	26.46 ± 7.36	952.35 ± 21.32	495.16 ± 12.33	29.65 ± 5.01

Values in each cell are presented as mean ± standard deviation. ‘-’ means compounds were not detected. ^a^ JYH refers to *Lonicerae japonicae* flos. ^b^ SYH refers to *Lonicerae flos*. ^c^ The amount of TFC is expressed as μg QE/mL of JYH or SYH extract. ^d^ ORAC and ABTS assays are expressed as μg TE/mL of JYH or SYH extract. After calculating the *p*-value between each two values using *t*-test, they were significantly different (*p* < 0.05) from each other.

## Data Availability

The original contributions presented in this study are included in the article. Further inquiries can be directed to the corresponding author.
